# Prescription of anxiolytics by other specialties: a survey of knowledge and attitudes

**DOI:** 10.1192/j.eurpsy.2023.1427

**Published:** 2023-07-19

**Authors:** B. Zineb, T. Aicha, K. Imane, S. Maria, E. O. Fatima

**Affiliations:** Faculty of Medecine and Pharmacy of Rabat, Ar-razi Psychiatric Hospital, Rabat, Morocco

## Abstract

**Introduction:**

Benzodiazepines (BZDs) have a broad spectrum of therapeutic effects, with undeniable efficacy and low toxicity/lethality. The high prevalence of anxiety and sleep disorders makes them one of the most prescribed drugs in the world.

We have chosen to focus on these drugs because of the interest they arouse nowadays due to the potential danger linked to their prolonged or abusive use, which can have major consequences on the state of health of populations.

**Objectives:**

The objective of this study is to evaluate the knowledge and attitudes of general practitioners in relation to the prescription of benzodiazepines, with a view to possibly proposing recommendations aimed at rationalizing the use of these drugs in Morocco.

**Methods:**

The method used is therefore the semi-structured interview, lasting on average 40 minutes, a fairly free method which allows the collection of valuable information.

This method is relevant for analysing the meaning that prescribers give to their practices, to highlight the knowledge and reference points from which they orient themselves and determine their practices. It allows for a “continuous process of verification and reformulation of hypotheses”. As the survey progresses, new questions can be addressed.

A questionnaire was therefore developed and used as the basis for the interview. It included practical questions relating to the activity of the doctors, their practice in consultation, their training, their management of psychiatric pathologies, their knowledge of benzodiazepines, and finally, their opinion on the overprescription of this molecule.

**Results:**

The survey was conducted among 10 general practitioners, 8 of whom prescribed benzodiazepines for the treatment of anxiety, insomnia and depression.

The first molecule prescribed was alprazolam, for an average duration of more than three months.

**Conclusions:**

To this end, several measures have been taken in some developed countries to regulate prescribing in order to improve proper use, control consumption and avoid misuse of these drugs.

**Disclosure of Interest:**

None Declared

